# Sensitive colorimetric assay using insulin G-quadruplex aptamer arrays on DNA nanotubes coupled with magnetic nanoparticles

**DOI:** 10.1098/rsos.171835

**Published:** 2018-03-21

**Authors:** A. Rafati, A. Zarrabi, S. Abediankenari, M. Aarabi, P. Gill

**Affiliations:** 1 Department of Biotechnology, Faculty of Advanced Science and Technology, University of Isfahan, Isfahan, Iran; 2 Nanomedicine Group, Immunogenetics Research Center, Mazandaran University of Medical Science, Sari, Iran; 3 Diabetes Research Center, Mazandaran University of Medical Science, Sari, Iran; 4 Ischemic Disorders Research Center, Golestan University of Medical Sciences, Gorgan, Iran

**Keywords:** G-quadruplex aptamer, MNP-coupled DNA nanotube, insulin colorimetric assay

## Abstract

Described here is a methodology for fabrication of a sensitive colorimetric nanoassay for measurement of insulin using G-quadruplex aptamer arrays on DNA nanotubes (DNTs) coupled with magnetic nanoparticles. The spectroscopic findings (e.g. visible spectra, velocity assay and limit of detection determination) indicated a highly sensitive performance of this new nanoassay in comparison to those results obtained from the insulin assay with non-arrayed aptamers. The clinical performance statistics (i.e. paired sample *t*-test, Bland–Altman plot and scatter diagram) from the newly developed assay and the enzyme-linked immunosorbent assay suggested its reliable precision and its acceptable repeatability for measurement of insulin in human sera. This is, to our knowledge, the first study for the application of magnetic nanoparticle-coupled DNTs for carrying G-quadruplex aptamers for detection of biomolecules (such as insulin) in human serum.

## 1. Introduction

Colorimetric assays contain a recognition part for trapping of a target molecule and a signal transduction part for reporting the presence of that trapped molecule [1,2]. Antibodies are the tools known as recognition elements for trapping of the target molecules with high specificity; however, their signal transduction parts are usually enzymes (such as horseradish peroxidase) with their sensitivity, stability and costs unfavourable in experimental conditions [3,4]. However, an enzyme-linked immunosorbent assay (ELISA) is the most commonly used method for quantitative detection of target molecules in clinical laboratories with its main reagent being the antibody and peroxidase enzyme-conjugate (e.g. horseradish peroxidase) for colorimetric assays [5,6].

Recently, nucleic acid-based recognition elements, i.e. aptamers have been attracting much attention as alternative candidates for the antibodies, with their conjugates with the enzymes being employed in colorimetric assays [7–10]. The aptamers are single-stranded DNA or RNA oligonucleotides that recognize their target molecules such as ions to macromolecules with high affinity and specificity [11–13]. Aptamers can switch to their specific folding by trapping their target molecules [14]. Particularly, aptamers have the capability of jointing to G-quadruplex DNAzymes via a helicase-dependent amplification with specific primers for enhancing the sensitivity of the enzyme-mediated analytical detection methods (aptazyme-linked oligonucleotide assay; ALONA) [15]; however, a label-free amplified DNA detection system based on an Exo III assisted strand-cleavage cycle was developed as a ligand-responsive quadruplex formation [16]. Also, the formation of G-quadruplex DNA could be detected through highly specific fluorescent probes [17,18].

There are some aptamers that included a G-rich sequence and these types of aptamers fold to a G-quadruplex structure after capturing their targets [19,20]. The G-quadruplex DNA aptamers were investigated against a variety of target molecules such as proteins [21–23]. This G-quadruplex structure plays a significant role for signal transduction from the specific detection of an analyte by aptamers [24,25]. By adding a hemin molecule to G-quadruplex-formed aptamers, peroxidase activity is then found similar to the function by peroxidase-mimicking DNAzymes [26].

**Figure 1.**
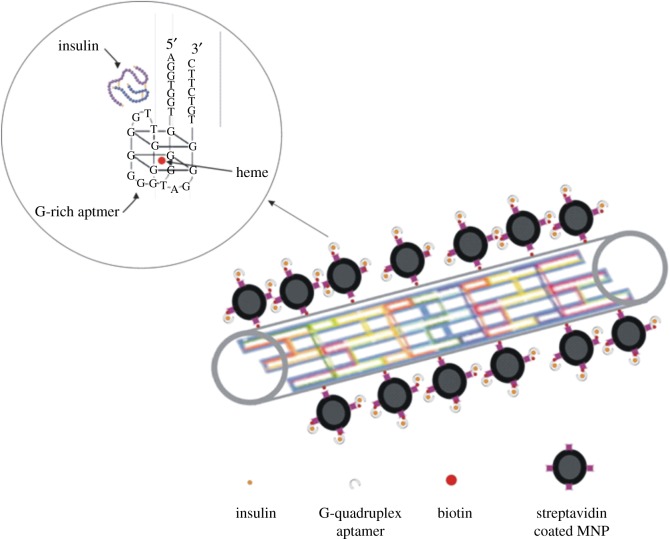
Schematic of the G-quadruplex insulin aptamer array on the surface of MNP-coupled DNA nanotubes. The inset graphically demonstrates the folding of a G-rich insulin aptamer after binding to its target and at the heme position.

Fabrication of nanoarrays from recognizing parts (e.g. aptamers or antibodies) for highly sensitive detection of target molecules has been technically used in developing colorimetric assays. For instance, carbon nanotubes have been previously applied for fabrication of nanoarrays of aptamers and antibodies in detection of biomolecules. Here, we introduced the capabilities of DNA nanotubes (DNTs) coupled to magnetic nanoparticles (MNPs) as chimeric or hybrid nanomaterials for developing a colorimetric assay for insulin via a nanoarray of G-rich aptamers (figure 1). The colorimetric assay then was compared with an insulin ELISA.

## 2. Material and methods

### 2.1. Chemicals

BIO-RP-purified DNA oligonucleotides (such as biotin-5′ GGTGGTGGGGGGGGTTGGTAGGGTGTCTTC3′, as a biotin-labelled G-quadruplex insulin aptamer [27], and DNT staples) were synthesized by Bioneer, Korea. In addition, to rule out non-specific insulin binding, a control 30 mer DNA oligonucleotide was synthesized by Bioneer with a random sequence (biotin-5' NNNNNNNNNNNNNNNNNNNNNNNNNNNNNN3′). M13mp18phage genome and T4 DNA ligase were purchased from New England Biolabs (Massachusetts, USA). Quantum Prep Freeze ‘N Squeeze DNA gel-extraction spin columns were obtained from Bio-Rad. Streptavidin (fluidMAG-Streptavidin) magnetic nanoparticles of 100 nm diameter size and a Magneto PURE-Micro separator were purchased from Chemicell (Germany). 3, 3′, 5, 5′ tetramethylbenzidine (TMB), hemin (Bioextra, from porcine), insulin and stop reagent for the TMB substrate were provided from Sigma, USA. Hemin stock solution (5 mM) was prepared in dimethyl sulfoxide (DMSO) (purchased from Bio Idea Company, Tehran, Iran), stored in the dark at – 20°C and diluted to the required concentration with buffer solution (20 mM Tris-HCl, 40 mM KCl, 200 mM NaCl, 0.06%(v/v) Triton X-100, pH 7.4). The desired concentration of insulin was dissolved in binding buffer (20 mM Tris-HCl, 140 mM NaCl, 5 mM KCl, 1 mM CaCl_2_ and 1 mM MgCl_2_, pH 7.4). The insulin ELISA kit was purchased from Demeditec, Germany.

### 2.2. Apparatuses

The thermal condition for self-assembly of the DNT in origami reaction was set using the Rotor Gene Q machine (QIAGEN, Germany). All the assays were performed using a 96-well immunoplate from SPL Life Science (Gyeonggi-do, Korea). For any level of heating interference a Master Cycler Personal (Eppendorf, Germany) was used. For shaking of the ELISA plates a rotator 430 from the Pole Idea Pars Company (Tehran, Iran) was used. Ultraviolet (UV)–visible absorption spectra were measured using a Spectrophotometer (JenWay 6505, UK).

### 2.3. Fabrication of insulin G-quadruplex aptamer arrays on magnetic nanoparticle-coupled DNA nanotubes

MNP-coupled DNTs have been fabricated according to the procedure published recently by Rafati *et al.* [28]. For this purpose, a biotinylated DNT was fabricated via an origami reaction prepared by combining 20 nM DNA scaffold M13mp18 single-stranded DNA and 100 nM of each staple oligonucleotide (table 1) that were diluted in 1× Tris base, acetic acid and EDTA buffer (40 mM Tris–acetic acid buffer (pH 8.0) and 12.5 mM magnesium acetate). The mixtures were kept at 95°C for 5 min and then annealed from 95°C to 20°C at a constant rate of –1°C min^−1^ in the thermocycler.

**Table 1.** Staple oligomer sequences for self-assembly of DNA nanotubes.

no.	sequence (5′–3′)
1	Bio-CCAACGTGCAGGTCATTCGTA
2	Bio-CACTATTCCGGTTCATGGTCG
3	Bio-TTCCAGTTCCCTTAAGCAGGC
4	Bio-GAGATAGGGTTGACGCGCGGGGAGAGGCGGT
5	Bio-ACGGCCAGTGCCTGTTTCCTG
6	CATGCCTCAAAGGGGCGCTCA
7	GAGGATCAAAGAACGTCGGGA
8	GGCAAAATTGGAACGCTGCAT
9	ATCATGGGCTCACAAATGAGTGAGCTAACTCAC
10	GGTACCGACGAGCCAGTGTAA
11	GAAAATCTTGCCCTCACCAGT
12	Bio-CATGCCTCAAAGGGGCGCTCA
13	Bio-TGTGAAATTGTTATCCTCATAGCAAGCTTG
14	ACAACATAGCTCGAGACTCTA
15	CAGCTGACTGTTTGCGAAATC
16	CTGGCCCTTGCCCCTAAATCAAAAGAATAGCCC
17	AGCCTGGCTTTCCAGTGGACT
18	GAGACGGCGTGCCAAAGAGTC
19	GTGGTTTTCGGCCAAGTGTTG
20	Bio-TTGCGTATTGGGGTTGCAGCA
21	Bio-ATTAATTGCGTTCGAAAAACCGTCTATCACG
22	Bio-CTGCCCGGGTGCCTATTCCAC
23	Bio-AACCTGTGCCATAAGGAAGAA
24	Bio-TAATGAATTCTTTTTCACCGC

For the fabrication of sturdier DNTs, the origami products were treated by a ligation process with T4 DNA ligase. The ligation–reaction mix was prepared containing 2 µl of 10× T4 DNA ligase reaction buffer, 10 µl of self-assembled DNTs, 2 µl of 50% polyethylene glycol and 1 µl of T4 DNA ligase enzyme. Finally, the ligation mix was incubated at 37°C for 1 h. The biotinylated DNTs were conjugated with 2.5 × 10^−3^ mg ml^−1^ streptavidin-coated MNPs and then incubated at 37°C for 5 min. Then the nanotubes were separated from the reaction materials via electrophoresis in 1% agarose gel. The attended bond on the gel for MNP-coupled DNTs was extracted by the use of quantum prep freeze N squeeze DNA gel extraction spin columns according to the manual instruction.

### 2.4. Colorimetric detection of insulin by G-rich aptamer arrays on magnetic nanoparticle-coupled DNA nanotubes

Two µM biotin-labelled G-rich aptamers were added to MNP-coupled DNTs and incubated at 37°C for 10 min. The mixture was placed in a Magneto PURE-Micro separator and washed twice with 5 mM Tris buffer to remove additional aptamer molecules. Then, 100 nM insulin in binding buffer was added and the solution was kept for 40 min at room temperature to allow formation of appropriate folding of the aptamer–insulin complexes. Up to 150 µl of 20 µM hemin solution in DMSO was added to the mixture, which was then kept at room temperature for 1 h to form the insulin–aptamer–hemin complexes. A 75 µl aliquot of TMB–H_2_O_2_ was added and incubated for 30 min at room temperature. Then, 75 µl of stop reagent (H_2_SO_4_) was added to the mixture and the colorimetric changes were recorded in the wavelength range of 350–600 nm using a UV–Vis spectrophotometer. Also, the mixture with no G-rich aptamer oligonucleotide was measured as the background using UV–Vis spectroscopy. For the kinetic assay, absorbance of the TMB chromogenic product was measured at a wavelength of 650 nm for 5 min. At the end of assays, 75 µl of stop reagent (H_2_SO_4_) was used to stop the enzymatic reaction and the absorbance was recorded at 450 nm.

### 2.5. Quantitative measurement of insulin via colorimetric assay

Two series of insulin with 1, 10, 100 and 200 nM concentrations in binding buffer were prepared and then added separately to 2 µM G-rich aptamer and the nanoarray mixture for comparing their sensitivities for colorimetric measurements of insulin. The mixtures were incubated at room temperature for 1 h. Of note, up to 150 µl of 20 µM hemin solution in DMSO was added to the mixture, which was kept at room temperature for 1 h to form the insulin–aptamer–hemin complex. A 75 µl aliquot of TMB–H_2_O_2_ was added and incubated for 30 min at room temperature. Then, 75 µl of stop reagent (H_2_SO_4_) was added to the mixture and colorimetric changes were measured at 450 nm using the UV–Vis spectrophotometer. Also, the standard curve and its regression line equation were plotted for measuring insulin concentrations.

### 2.6. Limit of detection determination

Limit of detection (LoD) is used to describe the smallest concentration of a measurement that can be reliably measured by an analytical procedure [29]. For this purpose, two series of 1 : 2 serial dilutions of insulin (from 6.25 to 0 µIU ml^−1^) in sera were checked by the newly described test.

### 2.7. Statistics for assessing agreement of the colorimetric assay and enzyme-linked immunosorbent assay for insulin detection

The capability of accurate insulin detection and quantification of the designed assay was checked via 50 human sera samples, by determining the amounts of human insulin in comparison to the ELISA method done routinely at the clinical laboratories. Following ethical approval of the study protocol from the ethical committee of Mazandaran University of Medical Sciences, Iran, the sera samples were collected from the clinical laboratory of Tooba Clinic Center, Sari, Iran. The sera were checked via insulin ELISA kit and tested by the aptamer-based colorimetric assay. For assessing agreement of the aptamer-based colorimetric assay with ELISA for insulin measurements in human sera, the paired *t*-test and Bland–Altman bias plots were calculated by the methodology described previously by Bland & Altman [30].

## 3. Results and discussion

### 3.1. Insulin colorimetric detection of nanoarray

**Figure 2.**
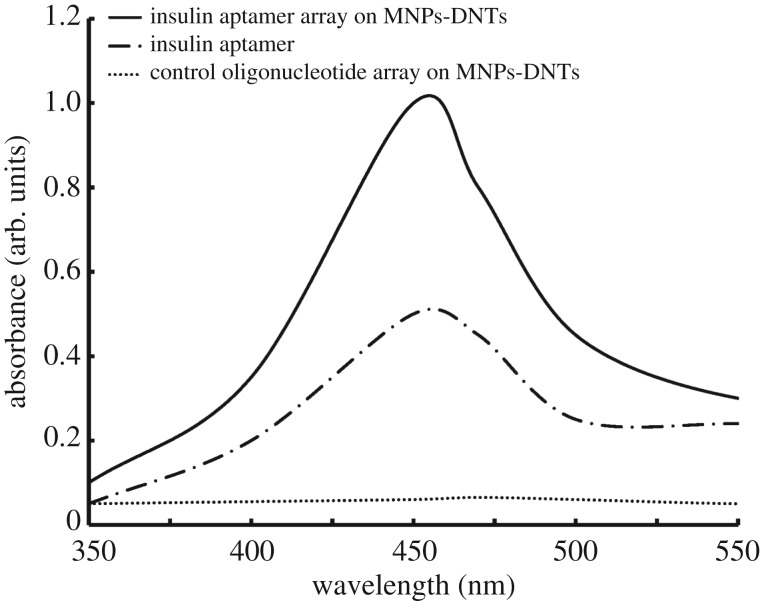
UV–Vis spectra of the insulin colorimetric assay using G-quadruplex insulin arrays on MNP-coupled DNA nanotubes (DNTs) in comparison to the results using G-quadruplex insulin aptamers and control random oligonucleotide on MNP-coupled DNTs.

The visible spectrum of the nanoarray-based insulin colorimetric assay showed a positive maximum at 450 nm, which was same as the result from an insulin G-rich aptamer assay. In addition, the nanoarray-based insulin colorimetric assay showed a higher absorbance at 450 nm in comparison to the result using insulin G-rich aptamer (figure 2). It seems the more colorimetric signal resulted because of the several G-rich aptamers carried by the MNP-coupled DNTs that enabled capture of several insulin molecules. This finding demonstrated that the DNT could be an alternative for the carbon nanotube previously used for carrying aptamers and antibodies for capturing more target biomolecules.

### 3.2. Peroxidase kinetic rates

**Figure 3.**
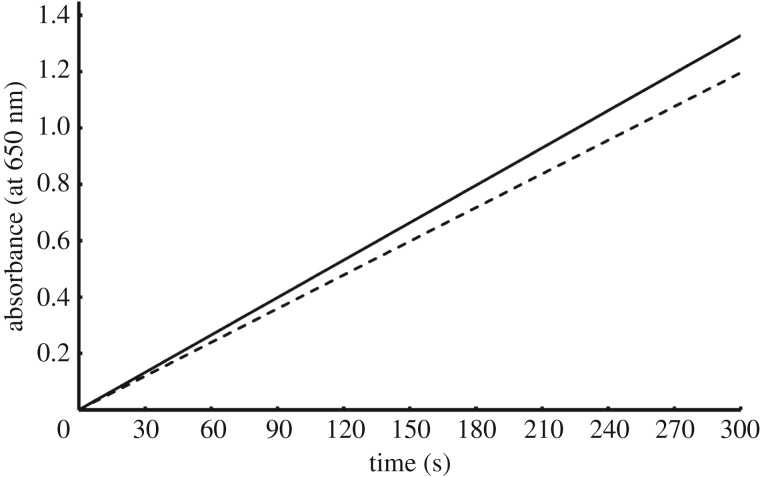
Steady-state kinetic assay of G-quadruplex insulin aptamers with peroxidase function in separate form and in the arrays on MNP-coupled DNA nanotubes; the absorbance changes at 650 nm during 5 min.

Steady-state kinetics of G-quadruplex insulin aptamer with peroxidase function in separate form and in the array on MNP-coupled DNTs were measured at the absorbance changes at 650 nm during 5 min (figure 3). The results demonstrated more velocity of the aptamer array (0.0033 OD s^−1^) than the results from aptamers (0.0026 OD s^−1^) separately. It seems the DNT could give a flexible area for positioning aptamers on it to perform capture selectively and make peroxidase function rapidly when compared with free aptamers.

### 3.3. Insulin measurement of nanoarrays

**Figure 4.**
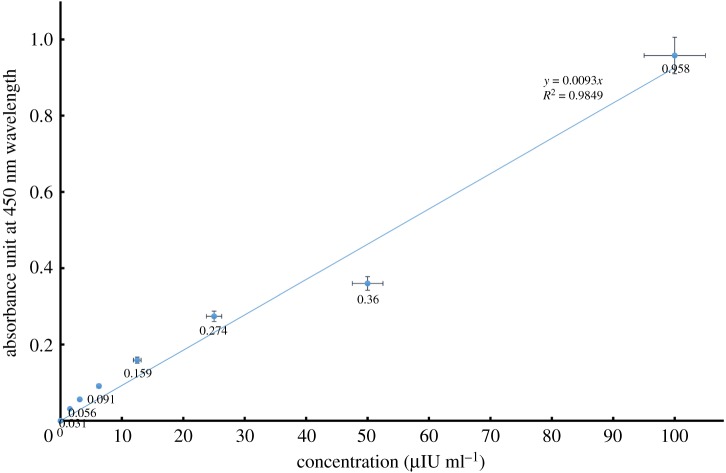
Standard curve of insulin detection via the aptamer array based on serial concentrations of insulin (1.56–100 µIU ml^−1^) with 2 µM G-rich aptamer.

The standard curve of insulin detection via the aptamer array based on serial concentrations of insulin (1.56–100 µIU ml^−1^) with 2 µM G-rich aptamer was obtained (figure 4). The trend of the curve indicated a linear relationship between insulin concentrations and the absorbances, thus the results could be employed in quantitative assay of insulin in the specimen.

### 3.4. Limit of detection

**Figure 5.**
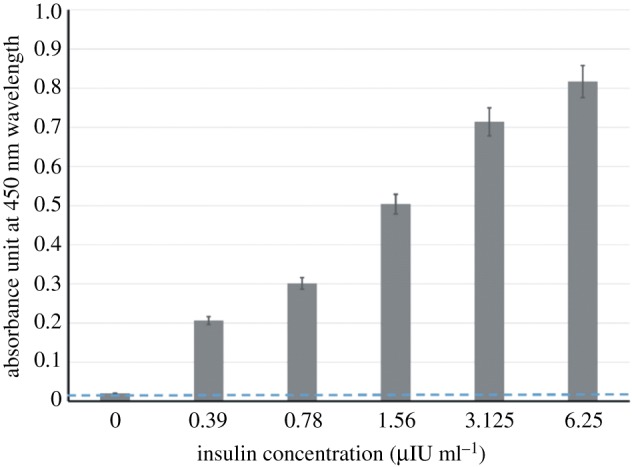
LoD (sensitivity) of the insulin via the aptamer arrays on MNP-coupled DNA nanotubes in comparison with the aptamers in separate form; non-specific binding (NSB) or background effect is shown with a dashed blue line nearly equal to 0.02 absorbance unit of zero standard sample (as negative control) with no insulin.

The minimum concentration of insulin that was measured by the manufacturer of the insulin ELISA kit was 6.25 µIU ml^−1^ (approx. 0.25 ng ml^−1^) in serum, whereas 0.39 µIU ml^−1^ (approx. 0.0156 ng ml^−1^) was measurable by this new colorimetric assay (figure 5); however, the range of our assay was standardized between 1.56 and 100 µIU ml^−1^. This finding showed that the LoD of the nanoarray-based colorimetric assay was less than that measured with the insulin ELISA kit. Therefore, the sensitivity of the developed nanoassay was more than that of the result from the routine ELISA for measuring insulin in serum specimens. In other words, by shaping the aptamer array on the DNT a highly sensitive signal could be obtained for the detection of insulin molecules, which cannot be achieved using an antibody horseradish peroxidase conjugate in the conventional ELISA method.

### 3.5. Performance characteristics

**Figure 6.**
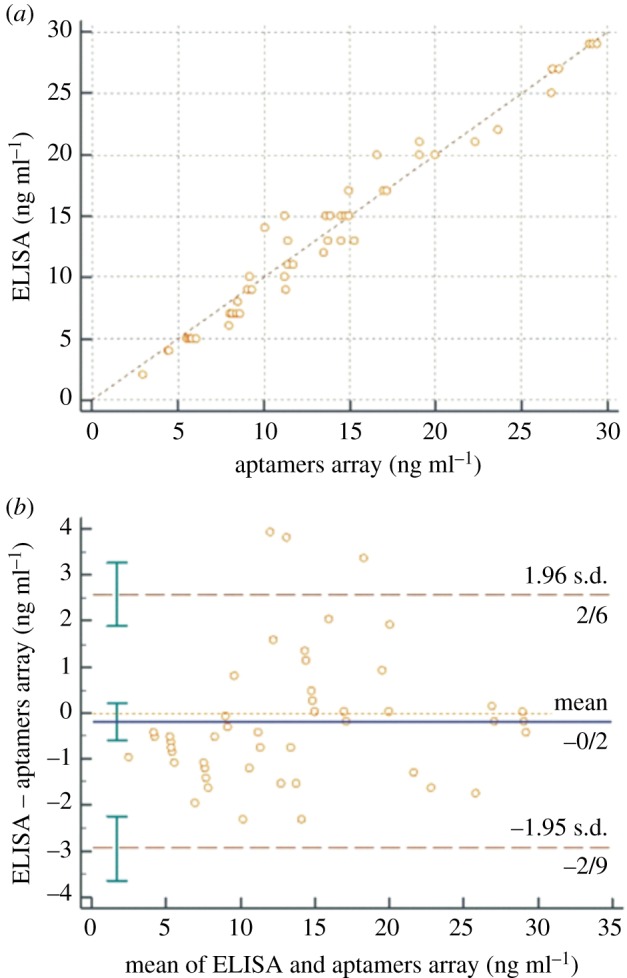
Scatter diagram (*a*) and Bland–Altman plot (*b*) of the insulin measurements (ng ml^−1^) in human sera by ELISA and the aptamer array colorimetric assay.

A paired samples *t*-test was included as given in table 2. The paired sample *t*-test indicated a mean difference of 0.18 between the two assays (*p*-value = 0.3713). Also, the Bland–Altman plot and scatter diagram of the two methods for insulin detection in human sera are shown in figure 6 and their summarized information has been presented in table 3.

**Table 2:** Paired sample *t*-test.

mean difference	0.18000
standard deviation of the mean difference	1.4106
standard error of the mean difference	0.1995
95% confidence interval	−0.2209 to 0.5809
test statistic *t*	0.902
degrees of freedom (d.f.)	49
two-tailed probability	*p*-value = 0.3713

**Table 3:** Bland–Altman plot.

differences between ELISA and the insulin aptamer array	
sample size	50
arithmetic mean	−0.1800
95% confidence interval (CI)	−0.5809 to 0.2209
*p*-value (*H*_0_: mean = 0)	0.3713
lower limit	−2.9448
95% CI	−3.6345 to −2.2551
upper limit	2.5848
95% CI	1.8951 to 3.2745
coefficient of repeatability	2.7596
95% CI	2.3090 to 3.434

In addition, the coefficient of repeatability of the assays was more than 2.5, which confirmed the suitability of variability between the two assays. These statistical findings showed that the new assay had acceptable selectivity and sensitivity in comparison to the routine ELISA test for insulin detection in serum; however, the reproducibility of the new assay was confirmed when compared with ELISA for measuring insulin in serum (table 3). Hence, the new insulin nanoassay suggested a cost-effective approach for employing DNT-based aptamer nanoarrays as an alternative to the conventional approach for employing monoclonal antibodies for insulin-measuring kits.

## 4. Conclusion

We have developed a colorimetric assay for human serum insulin using a nanoarray made from G-rich aptamers carried by MNP-coupled DNTs. This coupling of MNPs on DNTs has an advantage that several G-rich aptamers could be carried and a significant signal of detection created. Moreover, this approach is a more economical alternative as it might possibly involve a label-free approach (such as the use of selective DNA probes or nucleic acid amplifications). This colorimetric assay demonstrated a high sensitivity for insulin detection in specimens via shaping nanoarrays for obtaining peroxidase function after capturing insulin molecules using an MNP network of G-quadruplex aptamers on DNTs. This newly developed assay was compared to ELISA and ALONA for insulin measurements (table 4) according to their trapping and recognition elements. In addition, their dependencies on signal amplification, their costs and LoD were compared. This sensitive colorimetric assay matches the golden rules for the development of diagnostic applications such as low cost, user-friendly format such as a colorimetric assay, low sample consumption, easy adaptability to new targets, and ease of standardization and optimization.

**Table 4.** The insulin aptamers array in comparison to insulin ELISA and insulin ALONA.

analytical method	trapping element	recognition element	signal amplification need	transduction signal	cost	sensitivity (ng ml^−1^)	Ref no.
ELISA	monoclonal antibody	enzyme	no	colorimetric	expensive	0.07	[6]
ALONA	aptamer	HRP-mimicking DNAzyme	yes	colorimetric	less expensive	n.a.	[13]
insulin aptamer array	G-rich aptamer	peroxidase function	no	colorimetric	inexpensive	0.015	this study

## Supplementary Material

Supplementary Material# 1; Supplementary Material# 2; Supplementary Material# 3

## Data accessibility

The manuscript includes an electronic supplementary material. References are accessible through DOI numbers.
