# Supplementary material for: Sensitive colorimetric assay using insulin G-quadruplex aptamer arrays on DNA nanotubes coupled with magnetic nanoparticles
Source: R Soc Open Sci. 2018 Mar 21;5(3):171835. doi: 10.1098/rsos.171835 (PMC5882713; doi:10.1098/rsos.171835)
Supplement: Supplementary Material# 1; Supplementary Material# 2; Supplementary Material# 3 [file rsos171835supp1.docx]

Sensitive Colorimetric Assay using Insulin G-quadruplex Aptamer Arrays on DNA Nanotubes Coupled with Magnetic Nanoparticles

A. Rafati^1^, A. Zarrabi^1,*^, S. Abedian-Kenari^2^, M. Aarabi^3,4^, and P. Gill^2,3,*^

1. Department of Biotechnology, Faculty of Advanced Science and Technology, University of Isfahan, Isfahan, Iran

2. Nanomedicine Group, Immunogenetics Research Center, Mazandaran University of Medical Science, Sari, Iran

3. Diabetes Research Center, Mazandaran University of Medical Science, Sari, Iran

4. Ischemic Disorders Research Center, Golestan University of Medical Sciences, Gorgan, Iran

*Authors for correspondence

Ali Zarrabi; PhD

Department of Biotechnology, Faculty of Advanced Science and Technology, University of Isfahan, Isfahan, Iran

E-mail: a.zarrabi@ast.ui.ac.ir

Pooria Gill; PhD

Nanomedicine Group, Immunogenetics Research Center, Mazandaran University of

Medical Sciences, Sari, Iran

E-mail: pooriagill@yahoo.com; p.gill@mazums.ac.ir

Fax: +98-11-33261247; Tel: +98-11-33261247, +98-9127615950

Supplementary Materials

**Materials and Methods**

**Circular dichroism spectroscopy of G-rich aptamer-insulin interaction**

Twenty µM aptamer oligonucleotide was heated to 95 °C for 10 min to denaturize any intermolecular interaction and then kept on ice for preventing undesirable structural formation. Then, 100 nM of insulin at folding buffer were added and the mixture was incubated at room temperature for 1 h. Also, 20µM Hemin solution in DMSO and 20 µM were mixed at 25 mM Tris-HCl, pH 7 separately. The result was measured by CD spectroscope within 220-360 nm. CD parameters were set as the step resolution at 1 nm, speed 100 nm/min, accumulation at 1, response at 1 s, bond width at 1 nm and sensitivity 200 mdeg. Noise in the data was smoothed using software including the fast Fourier-transform noise reduction routine, which allows enhancement of most noisy spectra without distorting their peak shape.

**Atomic force microscopy of DNTs coupled with magnetic nanoparticles**

Five microliter of fabricated MNPs-coupled DNA nanotubes was immobilized on a mica surface for 4 hours at room temperature (25⁰C) to be dried. The samples were studied using contact mode with JPK-AFM, with 150 Hz IGain, 0.0048PGain, and 1.0 V set point via a JPK NanoWizard control. The cantilever was ACTA-10 probe model (material: silicon, N-type, 0.01–0.025 Ω/cm). Rough data were graphically processed with the JPK Nano analyser software.

**Results and Discussion**

**CD spectra of G-rich aptamer-insulin interaction**

CD spectra of G- rich aptamer showed negative (- 2500 ± 330 mdeg cm^2^/dmol) at 245 nm and positive maxima (2500 ± 240) mdeg cm^2^/dmol) at 275 nm after interaction with insulin. The molar ellipticities of G-rich aptamer showed negative (- 2400 ± 230 mdeg cm^2^/dmol) at 275 nm and positive maxima (3750 ± 220 mdeg cm^2^/dmol) at 335 nm after interaction with hemin. The CD spectra confirmed G-quadruplex formation structure at G-rich aptamer after interaction with insulin ^27^; however, the aptamer CD-spectrum could not confirmed such G-quadroplex structure after interaction with hemin (Supplementary Figure 1).

<< Supplementary Figure 1>>

**AFM of DNTs coupled with MNPs**

AFM result confirmed formation of nanoarray from MNPs at the exterior surface of DNTs (Supplementary Figure 2). The presence of these nanoarchitecture indicated that the staples selected for biotinylation were corrected and they could provide suitable positioning of streptavidin-coated MNPs at the exterior surface of DNTs.

<< Supplementary Figure 2>>

**Supplementary Figures**


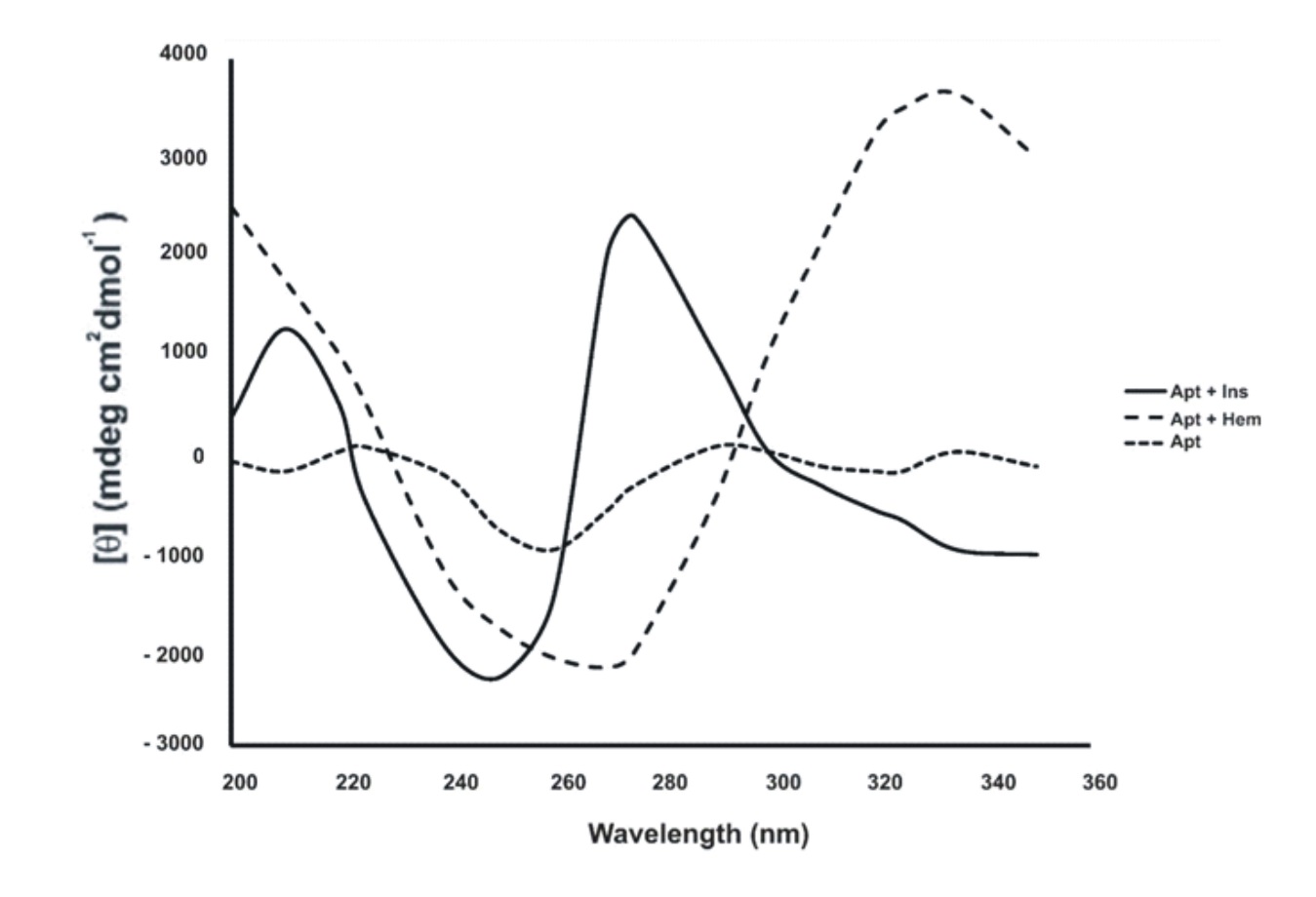


**Supplementary Figure 1.** CD spectra of G-rich aptamer-insulin interaction


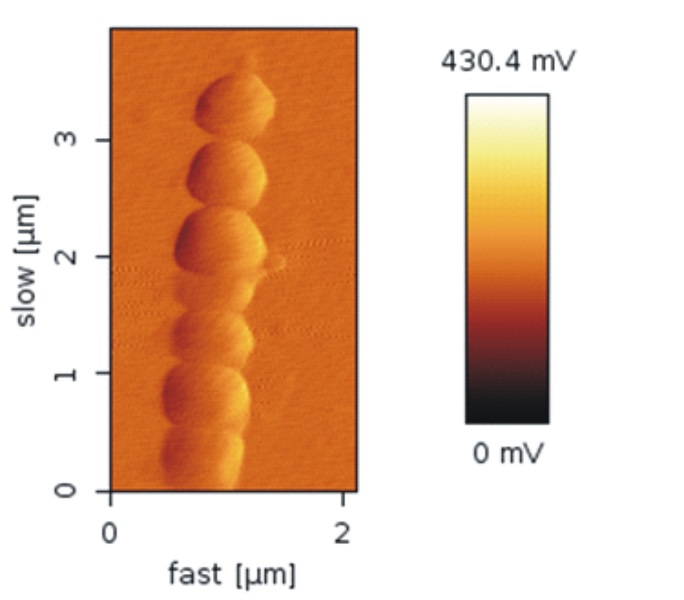


**Supplementary Figure 2.** AFM of DNT coupled with MNPs . The micrograph was gained and analyzed using Nano atomic force microscopy with 150 HzIGain, 0.0048 PGain, and 1.0 V set point via a JPK Nano Wizard control.


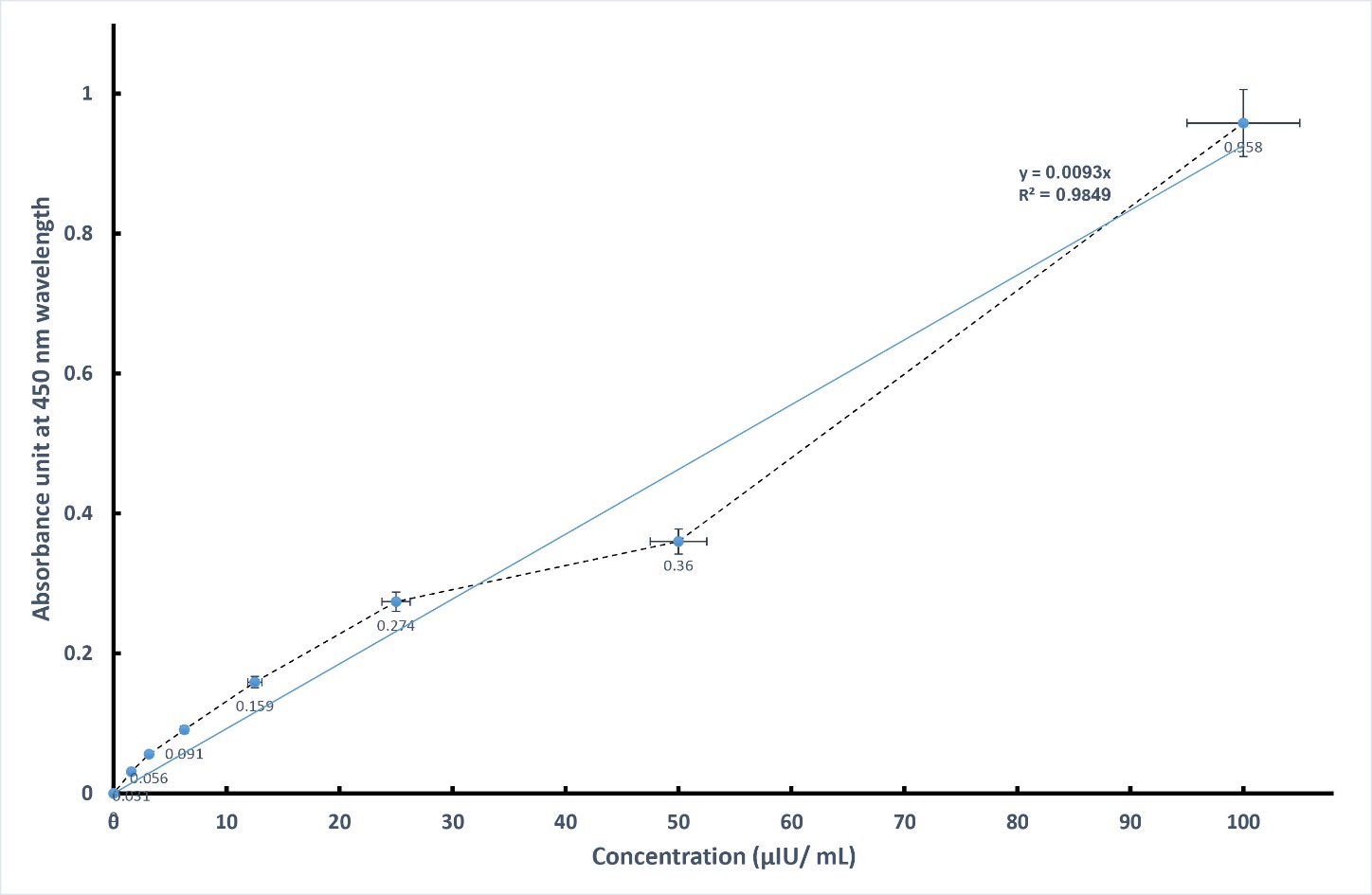


**Supplementary Figure 3.** Standard curve with a residual plot of insulin detection via the aptamers array based on a serial concentration of insulin (1.56-100 µIU/ml) with 2 µM G-rich aptamer
